# Supplementary material for: Content-rich biological network constructed by mining PubMed abstracts
Source: BMC Bioinformatics. 2004 Oct 8;5:147. doi: 10.1186/1471-2105-5-147 (PMC528731; doi:10.1186/1471-2105-5-147)
Supplement: Additional File 2 — The original results of the above study (non-essential files are deleted to keep the file size under the limit set by BMC bioinformatics). [file 1471-2105-5-147-S2.bz2 › chilibotAdditionalFile2/dip50/156ID8438581E558/html/N_M2.html]

 


 **N** and **M2** 
  
Found 5408 abstracts in PubMed, retrieved 50.  
 

 What does Google say? 
 PDF only 
| .edu only 

---

**Interactive relationship** (e.g. stimulation, inhibition, etc)

**Neutral relationship**- Allosteric site on muscarinic acetylcholine receptors identification of two amino acids in the muscarinic  **m2**  receptor that account entirely for the  **m2**  m5 subtype selectivities of some structurally diverse allosteric ligands in  **N**  methylscopolamine occupied receptors.  Ref: 12815157 Mol Pharmacol, 2003
- RESULTS After surgery, mean aortic valve area index AVAI improved from 0.57 cm2  **m2**  to 1.56 cm2  **m2** .Two groups of patients were identified Group A slow restenosers  **n**  = 16 and Group B rapid restenosers  **n**  = 6.  Ref: 12796014 Heart Surg Forum, 2003
- ThV was measured by ultrasound in adolescents aged 13 yr  **N**  = 1083 and 17 yr  **N**  = 1089 from 22 schools in 6 districts of eastern Slovakia and expressed as ml and ml  **m2**  body surface area.  Ref: 12803231 Endocr J, 2003
- Two epitopes have been identified recently to be responsible for the high affinity binding of alkane bisammonium and caracurine V type allosteric ligands to  **N**  methylscopolamine NMS occupied  **M2**  muscarinic acetylcholine receptors, relative to M5 receptors the amino acid  **M2**  Thr423 at the top of transmembrane region TM 7 and an epitope comprising the second extracellular loop o2 of the  **M2**  receptor including the flanking regions of TM4 and TM5.  Ref: 12815157 Mol Pharmacol, 2003

**Non-interactive relationship** (e.g. studied together, co-existance, homology, etc.)

- Although cysteine substitution mutations to residues in the  **N**  terminal half of the alpha subunit  **M2**  M3 loop dramatically impaired the gating efficacy, the same mutations exerted little effect when incorporated into corresponding positions of the beta subunit.  Ref: 12871591 J Neurochem, 2003
- 8 CdSO4 topology two new supramolecular isomers of  **M2**  bdc 2 L 2  **n**  coordination polymers.  Ref: 12841232 Chem Commun Camb , 2003
- INTERVENTIONS Shock trauma resuscitation protocol pulmonary artery catheter, gastric tonometry, urinary bladder pressure measurements, lactated Ringer infusion, packed red blood cell transfusion, and moderate inotrope support, as needed, in that sequence, to attain and maintain a DO2I greater than or equal to 600 mL min per  **m2**  16 months, ending January 001,  **n**  = 85 or a DO2I greater than or equal to 500 mL min per square meter 16 months, starting January 001,  **n**  = 71 for the first 24 hours in the ICU.Main Outcome Measures Lactated Ringer infusion volume liters at ICU admission, gastric partial carbon dioxide minus end tidal carbon dioxide GAPCO2, IAH urinary bladder pressure measurements >20 mm Hg, ACS urinary bladder pressure measurements >25 mm Hg with organ dysfunction, multiple organ failure, and mortality.  Ref: 12799335 Arch Surg, 2003
- In the narrow range of decreased mean ADC values 0.60 to 0.40 x 10 9  **m2**  x s 1, a 33% decrease in mean ADC is associated with a 122% increase in lactate  **N**  acetyl aspartate ratio.  Ref: 12817104 Stroke, 2003
- The unique six amino acid  **N**  terminal extension of Gqalpha when added to the  **N**  terminus of Gi1alpha neither prevented  **M2**  receptor coupling nor permitted M1 receptor coupling.  Ref: 12809511 Biochemistry, 2003
- DESIGN To this goal, in a large population  **n**  = 23 39 men, 98 women, age range 20 80 years, body mass index BMI range 26.4 4.3 kg  **m2**  of well nourished adults with total anterior pituitary deficit including severe GHD as shown by a GH peak below the 1st centile limit of normal response to GHRH ARG tests and or ITT we evaluated the diagnostic value of a single total IGF I measurement.  Ref: 12807504 Clin Endocrinol Oxf , 2003
- Patients were divided into two groups according to therapies given group I  **n**  = 30 no cardiac irradiation, median cumulative anthracycline dose 210 mg  **m2** .  Ref: 12805337 J Clin Oncol, 2003
- group II  **n**  = 9 irradiation in the cardiac region, median cumulative anthracycline dose 180 mg  **m2** .  Ref: 12805337 J Clin Oncol, 2003
- The filters were  **n** =10 PF1000N effective membrane surface 0.15  **m2** .  Ref: 12823420 Artif Organs, 2003
- Mean glomerular filtration rate decreased from 58 19 at 1 year  **n** =42 to 44 16 mL min 1.73  **m2**  body surface area at 10  **n** =33 years.  Ref: 12883184 Transplantation, 2003
- In addition, treatment with antioxidant  **N**  acetyl L cysteine inhibited anti Id induced apoptosis in G1 2 and  **M2**  10.  Ref: 12846046 Clin Exp Rheumatol< MedlineTA>Clin Exp Rheumatol, 2003
